# Presence of *Candida* and its associated factors in participants attending oral cancer screening in the lower northeastern area of Thailand

**DOI:** 10.1186/s12903-023-03198-2

**Published:** 2023-07-28

**Authors:** Krongkan Deeiam, Jintana Pankam, Vanvisa Sresumatchai, Patama Visedketkan, Warut Jindavech, Dulyapong Rungraungrayabkul, Kununya Pimolbutr, Boworn Klongnoi, Siribang-on Piboonniyom Khovidhunkit

**Affiliations:** 1 Department of Advanced General Dentistry, Faculty of Dentistry, Mahidol University, 6 Yothi Rd. Ratchathewi, Bangkok, 10400 Thailand; 2 Development of Disease Management Model for Oral Cancer with an Integration Network of Screening, Surveillance, and Treatment from Primary Care Unit to Tertiary Care in Nakhonratchasima Province Project, Faculty of Dentistry, Mahidol University, Bangkok, Thailand; 3 Department of Family Health, Faculty of Public Health, Mahidol University, Bangkok, Thailand; 4 Dental Department, Khamthaleso Hospital, Nakorn Ratchasima, Thailand; 5 Dental Department, Prathai Hospital, Nakorn Ratchasima, Thailand; 6 Department of Oral Medicine and Periodontology, Faculty of Dentistry, Mahidol University, Bangkok, Thailand; 7 Department of Oral and Maxillofacial Surgery, Faculty of Dentistry, Mahidol University, Bangkok, Thailand

**Keywords:** *Candida*, Oral potentially malignant disorders, Oral cancer, Dip-slide test, Oral cancer screening program, Cancer

## Abstract

**Background:**

Certain evidence indicated high prevalence of *Candida* in oral potentially malignant disorders (OPMDs) and oral cancer (OC). This study was aimed to investigate the presence of *Candida* and its associated factors in participants who attended the oral cancer screening program in the lower northeastern districts of Thailand.

**Methods:**

Convenient participants residing in the lower northeastern districts of Thailand who attended the oral cancer screening were enrolled. A questionnaire retrieving demographic characteristics, risk factors of oral cancer, and risk of having *Candida* was completed. Oral examination was performed by oral medicine specialists or oral surgeons. The participants were categorized into 4 groups according to their clinical diagnosis, namely normal oral mucosa (NOM), OPMDs/OC, non-OPMDs/OC and clinically suspected oral candidiasis (CSOC). Stimulated saliva flow rate was measured. Dip-slide test was performed in each participant to evaluate the presence of *Candida.* The levels of *Candida* were categorized into high and low levels according to the score received from the dip-slide test. Factors associated with high levels of *Candida* were identified using multivariate logistic regression analysis.

**Results:**

A total of 577 participants were recruited. High levels of *Candida* were found in 31.3%, 24.7%, 25.9% and 18.1% in the OPMDs/OC, the non-OPMDs/OC, the CSOC and the NOM groups, respectively. According to multivariate logistic regression analysis, age above 60 years, female gender, betel quid chewing habit, use of denture, hyposalivation, and being in the CSOC group were found to be significantly associated with high levels of *Candida.*

**Conclusion:**

Higher number of participants in the OPMDs/OC group was found to have high levels of *Candida*. Increasing age, female gender, betel quid chewing habit, use of denture, hyposalivation and having CSOC lesions were associated with high levels of *Candida.*

## Background

In 2020, the International Agency for Research on Cancer (IARC), World Health Organization (WHO), estimated that the number of new cancer cases in both men and women was 190,636 in Thailand and lip and oral cavity cancer was the 10th most common cancer [1]. It is well known that oral cancer (OC) is traditionally associated with tobacco consumption and alcohol abuse [2], however, in the northeastern part of Thailand, betel quid chewing habit also contributes to the development of OC [3].

In 2020, WHO collaborating Center for Oral Cancer and Precancer in the UK held a workshop related to terminology, definitions and classification of oral potentially malignant disorders (OPMDs) [4]. OPMDs are defined as any oral mucosal abnormality that is associated with a statistically increased risk of developing OC. These include leukoplakia, proliferative verrucous leukoplakia, erythroplakia, oral submucous fibrosis, oral lichen planus, actinic keratosis, palatal lesions in reverse smokers, oral lupus erythematosus, dyskeratosis congenita, oral lichenoid lesion and chronic graft-versus-host disease [4].

*Candida* are common microflora in the oral cavity and are usually regarded as being commensals. Nevertheless, in some conditions especially when the host immune system deteriorates, *Candida* can transform from a harmless commensal to a pathogenic organism causing oral mucosal infection [5]. Several factors are responsible for an increase in oral levels of *Candida* and these include increasing age, prolonged use of removable dentures, hyposalivation, long-term use of antibiotics or corticosteroids, uncontrolled diabetes mellitus, being hospitalized patients and human immunodeficiency virus (HIV) infection [6]. Tobacco smoking and alcohol consumption, major risk factors for OC, were also reported to be associated with an increased risk of *Candida* carriage [7, 8]. In addition, a study conducted in female participants in northern Thailand [9] showed that a higher percentage of *Candida albicans* carrier was observed in betel quid chewers compared to non-chewers (24% vs. 18%), although the difference did not reach statistical significance.

A few studies investigating the association between the presence of *Candida* and OC have also been reported [10, 11]. A matched case-control study conducted in Australia showed that the frequencies of *Candida* and oral colonization were significantly higher in patients with OC than those without OC [10]. Another study conducted in India also indicated a higher prevalence of *Candida* in the oral cavity of patients with OPMDs and OC compared to patients with apparently normal oral cavity [11]. A total of 95 subjects of which 25 served as normal controls, 30 as having OPMDs and 40 as having oral squamous cell carcinoma (OSCC) were included. Positive *Candida* growth on Sabouraud-dextrose agar medium was seen in 24%, 43% and 82% of individuals of the normal control, the OPMDs and the OSCC groups, respectively. The increased prevalence of positive *Candida* growth in OPMDs and OC implied that there might be an association between the presence of *Candida* and OC [11].

Given the limited research on the presence of *Candida* in patients with OPMDs and OC, this study was conducted to examine the prevalence of *Candida* and its associated factors in subjects who attended the OC screening in the lower northeastern districts of Thailand.

## Methods

### Ethical considerations

This study was approved by the Institutional Review Board, Faculty of Dentistry/Faculty of Pharmacy, Mahidol University (MU-DT/PY-IRB 2019/050.3107 and MU-DT/PY-IRB 2019/041.0307). This study was in full compliance with International Guideline for Human Research Protection including the Helsinki Declaration, the Belmont Report, CIOMS Guideline and the International Conference on Harmonization in Good Clinical Practice. All subjects provided a signed inform consent to participate in the study.

### Subject recruitment

The current cross-sectional study had 2 specific aims: (1) to report the prevalence of *Candida* in participants presented with or without oral lesions and (2) to investigate risk factors associated with the presence of high and low levels of *Candida.*

This study was a part of the project entitled “Development of disease management model for oral cancer with an integration network of screening, surveillance, and treatment in northeast health district” conducted for screening of oral cancer and OPMDs in the northeastern part of Thailand. A target population of 366,154 subjects was estimated from the registry of the Ministry of Public Health. The details regarding the screening has been previously published [12]. In brief, individuals aged ≥ 40 years old who lived in the 3 northeastern provinces of Buri Ram, Chaiyaphum, and Nakhon Ratchasima were selected.

**Fig. 1:**
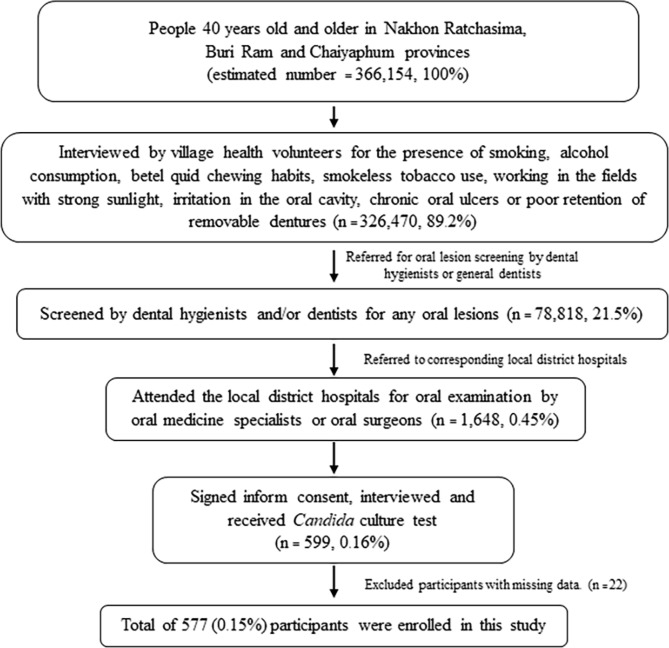
Flow chart of the study participants

The work flow of this research is shown in Fig. 1. First, subjects who were 40 years old and older (n = 326,470) were screened using a questionnaire by the village health volunteers (VHVs) whether they had any of the following risk factors for OC: tobacco smoking, alcohol consumption, betel quid chewing habit, smokeless tobacco use, working in the fields with strong sunlight, having irritation in the oral cavity, having chronic oral ulcers or wearing ill-fitting removable denture(s). Two researchers, S.P.K and BK, trained VHVs to initially investigate some certain types of oral lesions prior to the commencement of the study. If the subjects had 1 or more risk factor for OC, they were sent to the sub-district health promotion hospitals for the preliminary intra-oral screening of any suspicious OPMDs or OC. The screening was performed by dental hygienists or general dentists (n = 78,818). After the preliminary screening, if the subjects had any suspicious oral lesions, they were further sent to the district hospitals (n = 1,648). The dental hygienists and general dentists also eliminated the irritation in the oral cavity which might cause some oral lesions prior to the referral to district hospitals.

At the district hospitals, these subjects were asked to participate in the study. One of the study dentists (K.D.) explained the project and the informed consent was obtained from each participant. Exclusion criteria were subjects who were known cases or were in the process of the treatment of OC prior to the study recruitment, subjects with severe systemic diseases such as cancers elsewhere, severe blood diseases, uncontrolled diabetes or severe cardiac problems, subjects who received systemic or topical corticosteroid, antifungal medication, antibiotics, immunosuppressive drugs or used antiseptic mouth wash within 7 days prior to the saliva collection for *Candida* culture. Subjects with communication problems or those declined to participate were also excluded. Saliva collection was then performed followed by a thorough intra-oral examination by board-certified oral medicine specialists or board-certified oral surgeons. Clinical diagnosis was recorded, and incisional or excisional biopsies were performed for definitive diagnosis in certain cases if necessary.

The participants were separated into 4 groups including normal oral mucosa (NOM), OPMDs/OC, non-OPMDs/OC, and clinically suspected oral candidiasis (CSOC) groups. The participants in the NOM group were those with no visible oral lesions. The participants in the OPMDs/OC group were participants with visible OPMDs or OC. The clinical diagnosis of OPMDs were based on a consensus report from an international seminar on nomenclature and classification, convened by WHO Collaborating Center for Oral Cancer [4] including oral leukoplakia, oral erythroplakia, oral erythroleukoplakia, oral submucous fibrosis, oral lichen planus, oral lichenoid reaction, oral lupus erythematosus and actinic cheilitis. The non-OPMDs/OC group consisted of participants with benign or reactive oral lesions including irritation fibroma, giant cell fibroma, pyogenic granuloma, squamous papilloma, inflamed mucosa and frictional keratosis. The CSOC group composed of participants with suspicious oral lesions which contained *Candida* including pseudomembranous candidiasis, erythematous candidiasis, *Candida* associated denture stomatitis, median rhomboid glossitis, atrophic glossitis and angular cheilitis.

### Questionnaire

The questionnaire was composed of demographic data, systemic diseases, risk factors for OC and questions to evaluate whether the patients might have dry mouth. The xerostomia questions used in this study were modified from the xerostomia questions proposed by Torres et al. [13]. These 5 questions included (1) Do you feel that your mouth is dry? (2) Do you need to sip liquids to aid in swallowing dry food? (3) Do you feel uncomfortable during the day because your mouth is dry? (4) Do you have difficulty speaking due to oral dryness? (5) Do you have problems in tasting food? An affirmative response to at least one of the above five questions was defined as xerostomia.

Those who had never smoked or had stopped smoking for more than 12 months prior to the interview were classified as “not smoked” [10]. For betel quid chewing habits, use of smokeless tobacco, and alcohol intake, if the participants had never have or had stopped such activities for more than 12 months prior to the interview, they were similarly classified as participants who did not have such activities.

### Salivary flow rate measurement

A stimulated salivary flow rate was evaluated by a spit technique. The subjects were asked to chew a piece of paraffin for 5 min and spit their saliva into a cup. A 5-ml syringe was used to measure the amount of saliva and the salivary flow rate was calculated. According to the executive summary of a report from the American Dental Association Council on Scientific Affairs (2014), stimulated salivary flow rate of 0.7 milliliter per minute or less is defined as hyposalivation [14].

### Evaluation of ***Candida***

*Candida* were examined by the modified dip-slide test [15]. In the present study, the stimulated whole saliva sample was collected and subsequently poured over the surface of a dip-slide containing selective media for *Candida* (Sabouraud dextrose agar). All agar plates and plastic tubes containing dip-slides were incubated at 37 °C for 48 to 72 h in a 5% CO_2_ incubator. The density of the growth of *Candida* was scored by comparison with a chart provided with the test [15]. According to the score chart, levels of *Candida* were classified as 1, 2, 3 and 4 when the microorganisms were < 10^2^, >10^2^-< 10^3^, >10^3^-<10^4^ and > 10^4^ CFU per milliliter, respectively. In this study, the levels of *Candida* were categorized as low and high levels based on the dip-slide scores. Scores of 3 and 4 were considered as high levels of *Candida* with the estimated number of more than 1,000 CFU per milliliter. Scores of 0, 1 and 2 were classified as low levels (*Candida* colonies of less than 1,000 CFU per milliliter).

### Statistical analysis

Statistical analyses were performed using SPSS version 18.0 (SPSS Inc., Chicago, IL). Demographic data and the prevalence of *Candida* were described using frequency, percentage, mean, standard deviation, median and interquartile range according to the distribution of data. Demographic data, baseline characteristics, risk factors of OC and the presence of hyposalivation were compared using Chi-square tests for categorical variables. Kruskal-Wallis test followed by a post-hoc test for multiple comparisons was used for continuous nonparametric variables. Univariate and multivariate logistic regression analyses were performed to investigate associated risk factors of the presence of high levels of *Candida* in this population. The following covariates were included: age, gender, smoking status, betel quid chewing habits, use of smokeless tobacco, alcohol consumption, working in the fields with strong sunlight, history of medical illness, history of diabetes mellitus, denture status, presence of xerostomia or hyposalivation. All potential factors were adjusted and then removed one by one in a backward stepwise analysis. The probability of factor entry and removal was set to 0.05 and 0.1, respectively. All statistical tests were two-tailed and p-value < 0.05 was considered statistically significant.

## Results

### Participant demographics

A total of 577 participants were enrolled in this study (132, 165, 196 and 84 participants were in the NOM, OPMDs/OC, non-OPMD and CSOC groups, respectively). In the OPMDs/OC group, 159 participants presented with OPMDs and 6 participants presented with OC. Clinical characteristics of the four groups were relatively comparable as shown in Table 1. The mean age of participants was 65.69 years (SD = 9.73), with a range of 40 to 95 years. No significant differences in age, smokeless tobacco use, alcohol consumption, working in the fields with strong sunlight, history of medical illness and hyposalivation were found among the four groups. Non-OPMDs/OC lesions were more common in male participants (p < 0.001). The frequency of female gender, being current betel quid chewers and having diabetes mellitus were significantly higher in the OPMDs/OC group compared to the other groups (p < 0.05). The frequency of current smokers was found to be more common in the non-OPMDs/OC group than the other groups (p < 0.001).

**Table 1:** Demographic data of the participants (N = 577)

Variables	**Clinical diagnosis**					*p value*
	NOM	**OPMDs/OC**	Non-OPMDs/OC	**CSOC**	Total	
	(n = 132)	(n = 165)	(n = 196)	(n = 84)	(n = 577)	
	**n (%)**	**n (%)**	**n (%)**	**n (%)**	**n (%)**	
**Age (years)**						
40–60	47 (27.5)	42 (24.6)	63 (36.8)	19 (11.1)	171 (100)	0.11
61–69	48 (24.4)	58 (29.4)	65 (33.0)	26 (13.2)	197 (100)	
≥ 70	37 (17.7)	65 (31.1)	68 (32.5)	39 (18.7)	209 (100)	
**Gender**						< 0.001*
Male	44 (23.4)	43 (22.9)	87 (46.3)	14 (7.4)	188 (100)	
Female	88 (22.6)	122 (31.4)	109 (28.0)	70 (18.0)	389 (100)	
**Smoking status**						
Non-smoker	117 (24.5)	135 (28.2)	147 (30.8)	79 (16.5)	478 (100)	< 0.001*
Current smoker	15 (15.2)	30 (30.3)	49 (49.5)	5 (5.1)	99 (100)	
**Use of smokeless tobacco**						
Non-user	123 (24.3)	140 (27.6)	172 (33.9)	72 (14.2)	507 (100)	0.153
Current user	9 (12.9)	25 (35.7)	24 (34.3)	12 (17.1)	70 (100)	
**Alcohol consumption**						
Non-drinker	109 (23.5)	137 (29.6)	145 (31.3)	72 (15.6)	463 (100)	0.053
Current alcohol drinker	23 (20.2)	28 (24.6)	51 (44.7)	12 (10.5)	114 (100)	
**Betel quid chewing habit**						
Non-chewer	105 (27.3)	85 (22.1)	149 (38.8)	45 (11.7)	384 (100)	< 0.001*
Current chewer	27 (14.0)	80 (41.5)	47 (24.4)	39 (20.2)	193 (100)	
**Working in the fields with strong sunlight**						
No	58 (21.6)	83 (30.9)	84 (31.2)	44 (16.4)	269 (100)	0.319
Yes	74 (24.0)	82 (26.6)	112 (36.4)	40 (13.0)	308 (100)	
**Having irritation in the oral cavity**						
No	116 (26.0)	130 (29.1)	155 (34.7)	46 (10.3)	447 (100)	< 0.001*
Yes	16 (12.3)	35 (26.9)	41 (31.5)	38 (29.2)	130 (100)	
**History of medical illness**						
No	53 (21.4)	67 (27.0)	89 (35.9)	39 (15.7)	248 (100)	0.639
Yes	79 (24.0)	98 (29.8)	107 (32.5)	45 (13.7)	329 (100)	
**Diabetes Mellitus**						0.024*
No	113 (24.6)	119 (25.9)	161 (35.0)	67 (14.6)	460 (100)	
Yes	19 (16.2)	46 (39.3)	35 (29.9)	17 (14.5)	117 (100)	
**Use of denture**						
No	101 (20.0)	148 (29.2)	177 (35.0)	80 (15.8)	506 (100)	< 0.001*
Yes	31 (43.7)	17 (23.9)	19 (26.8)	4 (5.6)	71 (100)	
**Xerostomia**						0.06
No	79 (24.2)	101 (31.0)	113 (34.7)	33 (10.1)	326 (100)	
Yes	53 (21.1)	64 (25.5)	83 (33.1)	51 (20.3)	251 (100)	
**Saliva flow rate (ml/min)**						
Mean ± SD	0.72 ± 0.54	0.69 ± 0.67	0.67 ± 0.48	0.55 ± 0.45	0.67 ± 0.55	
Median	0.6	0.48	0.51	0.4	0.5	0.081^#^
(Interquartile range)	(0.32,1.03)	(0.24,0.92)	(0.30,0.99)	(0.20,0.78)	(0.25,0.95)	
**Hyposalivation (< 0.7 ml/min)**						
No	54 (24.5)	62 (28.2)	81 (36.8)	23 (10.5)	220 (100)	0.144
Yes	78 (21.8)	103 (28.9)	115 (32.2)	61 (17.1)	357 (100)	
**Levels of** ***Candida***						
0	13 (16.5)	26 (32.9)	30 (38.0)	10 (12.7)	79 (100)	
1	57 (26.4)	50 (23.1)	91 (42.1)	18 (8.3)	216 (100)	
2	32 (27.6)	37 (31.9)	34 (29.3)	13 (11.2)	116 (100)	
3	12 (17.1)	19 (27.1)	24 (34.3)	15 (21.4)	70 (100)	
4	18 (18.8)	33 (34.4)	17 (17.7)	28 (29.2)	96 (100)	

*Bold values denote statistical significance at the p < 0.05 level.; ^#^Kruskal Willis Test

### Prevalence of ***Candida*** in participants according to oral lesions

The levels of *Candida* were categorized into 2 levels: the low (< 1000 CFU per milliliter) and the high (> 1000 CFU per milliliter) levels. The high levels of *Candida* were found in 31.3%, 25.9%, 24.7% and 18.1% of the OPMDs/OC, CSOC, Non-OPMDs/OC and NOM groups, respectively (Table 2). Statistically different percentages of participants with high levels of Candida among four different groups of oral lesions were observed (p < 0.001). The high *Candida* levels were more predominant among those with OPMDs/OC (31.3%) whereas the majority of patients with low *Candida* levels were in the non-OPMDs/OC group (37.7%). In addition, it was found that the median scores of *Candida* levels were significantly different between (1) the non-OPMDs/OC group compared to the CSOC group (p<0.05) and (2) the NOM group compared to the CSOC group (Table 2; p < 0.05).

**Table 2:** Prevalence of *Candida* in participants according to oral lesions

Clinical diagnosis	Number of patients (n, %)		*p* value	*Candida* levels		*p* value^#^
	**Low levels** **(< 1,000 CFU/ml)**	**High levels** **(≥ 1,000 CFU/ml)**		**Mean score ± SD**	**Median (IQR)**	
NOM (n = 132)	102 (24.8)	30 (18.1)	< 0.001*	1.73 ± 1.18	1 (1,2)^a^	< 0.001*
OPMDs/OC (n = 165)	113 (27.5)	52 (31.3)		1.90 ± 1.36	2 (1,3)	
Non-OPMDs/OC (n = 196)	155 (37.7)	41 (24.7)		1.53 ± 1.15	1 (1,2)^b^	
CSOC (n = 84)	41 (10.0)	43 (25.9)		2.39 ± 1.44	3 (1,4)^a,b^	
Total	411 (100)	166 (100)		1.82 ± 0.92	1 (1,3)	

*Bold values denote statistical significance at the p < 0.05 level

Abbreviations: Normal oral mucosa; NOM, Oral potentially malignant disorders; OPMDs, Oral cancer; OC, Clinically suspected oral candidiasis; CSOC, Interquartile range; IQR.

^#^Kruskal-Wallis test

^a^ Statistical difference between the CSOC group and the NOM group

^b^ Statistical difference between the CSOC group and the non-OPMDs/OC

### Association of ***Candida*** in Thai patients with risk factors of OC

Several factors were found to be associated with *Candida* in the saliva. Univariate logistic regression analyses indicated that participants aged between 61 and 69 years (OR = 2.11, 95%CI 1.25–3.55), aged ≥ 70 years (OR = 3.90, 95%CI 2.40–6.43), female gender (OR = 5.05, 95%CI 3.04–8.38), current smoker (OR = 0.20, 95%CI 0.10–0.42), current alcohol drinker (OR = 0.46, 95%CI 0.27–0.78), current betel quid chewer ((OR = 4.62, 95%CI 3.14–6.77), working in the fields with strong sunlight (OR = 0.51, 95%CI 0.36–0.74), having hyposalivation (OR = 2.80, 95%CI 1.85–4.23) and being in the CSOC group (OR = 3.57, 95%CI 1.98–6.43) were statistically significantly associated with levels of *Candida* (p < 0.05) (Table 3). In the multivariate logistic regression analyses, it was found that age 61–69 years old (OR = 1.87; 95%CI 1.06–3.30), ≥ 70 years old (OR = 2.65; 95%CI 1.52–4.62), female gender (OR = 2.62; 95%CI 1.47–4.65), being current betel quid chewer (OR = 2.82; 95%CI 1.77–4.51), use of denture (OR = 1.96; 95%CI 1.07–3.61), presence of hyposalivation (OR = 2.27; 95%CI 1.44–3.60) and being in the CSOC group (OR = 2.69 95%CI 1.39–5.22) were significantly associated with high levels of *Candida.*

**Table 3:** Univariate and multivariate logistic regression analyses to assess associated factors with the level of *Candida*.

Variable	*Candida*		High levels			
	**Low levels** **(n = 411)**	**High levels** **(n = 166)**				
	**n (%)**	**n (%)**	**OR (unadjusted)** **(95% CI)**	***p value***	**OR (adjusted)** **(95% CI)**	***p value***
**Age (years)**						
40–60	145 (84.8)	26 (15.2)	1		1	
61–69	143 (72.6)	54 (27.4)	2.11 (1.25–3.55)	**0.05**	1.87 (1.06–3.30)	0.03*
≥ 70	123 (58.9)	86 (41.1)	3.90 (2.40–6.43)	< 0.001*	2.65 (1.52–4.62)	< 0.001*
**Gender**						
Male	168 (89.4)	20 (10.6)	1		1	
Female	243 (62.5)	146 (37.5)	5.05 (3.04–8.38)	< 0.001*	2.62 (1.47–4.65)	< 0.001*
**Smoking status**						
Non-smoker	321 (67.2)	157 (32.8)	1		1	
Current smoker	90 (90.9)	9 (9.1)	0.20 (0.10–0.42)	< 0.001*	0.72 (0.31–1.65)	0.439
**Use of smokeless tobacco**						
Non-user	366 (72.2)	141 (27.8)	1		1	
Current user	45 (64.3)	25 (35.7)	1.44 (0.85–2.44)	0.171	1.40 (0.73–2.68)	0.311
**Alcohol consumption**						
Non-drinker	317 (68.5)	146 (31.5)	1		1	
Current alcohol drinker	94 (82.5)	20 (17.5)	0.46 (0.27–0.78)	0.03*	1.05 (0.55–1.98)	0.891
**Betel quid chewing habit**						
Non-chewer	315 (82.0)	69 (18.0)	1		1	
Current chewer	96 (49.7)	97 (50.3)	4.62 (3.14–6.77)	< 0.001*	2.82 (1.77–4.51)	< 0.001*
**Working in the fields with strong sunlight**						
No	172 (63.9)	97 (36.1)	1		1	
Yes	239 (77.6)	69 (22.4)	0.51 (0.36–0.74)	< 0.001*	0.86 (0.56–1.31)	0.476
**Having irritation in the oral cavity**						
No	325 (72.7)	122 (27.3)	1		1	
Yes	86 (66.2)	44 (33.8)	1.36 (0.90–2.07)	0.146	1.22 (0.71–2.07)	0.473
**History of medical illness**						
No	185 (74.6)	63 (25.4)	1		1	
Yes	226 (68.7)	103 (31.3)	1.34 (0.93–1.94)	0.121	1.48 (0.93–2.36)	0.102
**Diabetes mellitus**						
No	328 (71.3)	132 (28.7)	1		1	
Yes	83 (70.9)	34 (29.1)	1.02 (0.65–1.59)	0.938	0.77 (0.47–1.27)	0.312
**Use of denture**						
No	364 (71.9)	142 (28.1)	1		1	
Yes	47 (66.2)	24 (33.8)	1.31 (0.77–2.22)	0.317	1.96 (1.07–3.61)	0.03*
**Xerostomia**						
No	241 (73.9)	85 (26.1)	1		1	
Yes	170 (67.7)	81 (32.3)	1.35 (0.94–1.94)	0.103	1.24 (0.81–1.91)	0.319
**Hyposalivation (< 0.7 ml/min)**						
No	183 (83.2)	37 (16.8)	1		1	
Yes	228 (63.9)	129 (36.1)	2.80 (1.85–4.23)	< 0.001*	2.27 (1.44–3.60)	< 0.001*
**Clinical diagnosis**						
NOM	102 (77.3)	30 (22.7)	1		1	
OPMDs/OC	113 (68.5)	52 (31.5)	1.57 (0.93–2.64)	0.093	1.08 (0.60–1.96)	0.803
Non-OPMDs/OC	155 (79.1)	41 (20.9)	0.90 (0.53–1.53)	0.697	0.96 (0.53–1.74)	0.893
CSOC	41 (48.8)	43 (51.2)	3.57 (1.98–6.43)	< 0.001*	2.69 (1.39–5.22)	0.003*

*Bold values denote statistical significance at the p < 0.05 level.

*Adjust for age, gender, smoking, alcohol consumption, betel quid chewing, exposure to sunlight, irritation, medical history, diabetes mellitus, use of denture, xerostomia, hyposalivation and clinical diagnosis.

### Relationship between the xerostomia questionnaire and the level of ***Candida***

We further investigated whether there were any differences in the responses to the xerostomia questionnaire (5 items) among those with the low and high levels of *Candida*. There were no significant differences in the responses to items 1–4 (Table 4). Interestingly, there was a significantly higher number of participants who experienced food tasting difficulties in the high level of *Candida* group (25.90%) compared to the lower level group (15.82%, p = 0.005).

**Table 4:** Comparison of responses on the questionnaire assessing xerostomia between the two groups with low and high levels of *Candida*

Questions	Participants with low levels of *Candida* (n = 411)	Participants with high levels of *Candida* (n = 166)	*p value*
	n (%)	n (%)	
**Do you feel that your mouth is dry?**			0.545
No	283 (68.9)	110 (66.3)	
Yes	128 (31.1)	56 (33.7)	
**Do you need to sip liquids to aid in swallowing dry food?**			1.63
No	315 (76.6)	118 (71.1)	
Yes	96 (23.4)	48 (28.9)	
**Do you feel uncomfortable during the day because your mouth is dry?**			0.557
No	345 (83.9)	136 (81.9)	
Yes	66 (16.1)	30 (18.1)	
**Do you have difficulty speaking due to oral dryness?**			0.22
No	360 (87.6)	139 (83.7)	
Yes	51 (12.4)	27 (16.3)	
**Do you have problems in tasting food?**			0.005*
No	346 (84.2)	123 (74.1)	
Yes	65 (15.8)	43 (25.9)	

*Bold values denote statistical significance at the p < 0.05 level

## Discussion

This is a large population-based study investigating the presence of *Candida* in saliva of subjects who attended the largest to date OPMDs and OC screening program in the northeastern districts of Thailand. High levels of *Candida* were more prominent in participants in the OPMDs/OC group. Increasing age, female gender, betel quid chewing habits, use of denture, hyposalivation and having CSOC lesions were associated with the high levels of *Candida.*

*Candida* is a normal commensal organism of the mouth and generally causes no complication in healthy people. Carriage rates in the general population have been reported ranging from 3 to 75% [16]. Previous studies have reported conflicting results on the association of *Candida* and OPMDs and OC. Some studies demonstrated the high prevalence of oral *Candida* carriage among patients with OPMDs and OC [10, 17], but the others did not find any association between *Candida* carriage and OPMDs [18, 19]. The inconsistent findings probably resulted from various methods used to isolate *Candida* from the oral cavity, different study populations, and the cut-off values for low or high levels of *Candida.*

Our study showed that several factors were significantly associated with the high levels of *Candida* in the saliva. Ages of 61–69 years old and older than 70 years old increased the risk of having high levels of *Candida* by 1.87 and 2.65 times, respectively. Female gender also increased the risk of high *Candida* levels by 2.62 times. These associations have been reported previously [20, 21].

A large number of participants in this study especially in the OPMDs/OC group were betel quid chewers. In the present study, participants with betel quid chewing habit were almost 3 times more likely to have high levels of *Candida* than those who had never chewed. The mechanisms of betel quid-induced oral carcinogenesis are currently unknown. However, the alterations appear to be linked to an oral microbiome problem, with lower bacterial numbers critical for maintaining homeostasis. OC appears to be linked to dysregulation of the immunological microenvironment caused by disturbance of the normal oral microflora and an increase in pathogenic microorganisms [22]. This present study was the first study reporting the association of betel quid chewing and high levels of *Candida*. Some studies did not find significant difference of *Candida* carriage between chewers and non-chewers nor the association between being *Candida* carriage and being betel quid chewers [9, 23–25]. A study conducted in Saudi Arabia revealed that oral *Candida* was isolated from 73.1% in individuals chewing betel quid with tobacco, 72.4% in individuals chewing betel quid without tobacco and 61% of non-chewers. It was concluded that chewing betel quid with or without tobacco does not influence oral *Candida* carriage [23]. Two studies conducted in Pakistan and Cambodia also reported no significant difference in the *Candida* carriage rate of the *Candida* isolated between betel quid chewers and non-chewers [24, 25]. A study conducted in northern Thailand also reported no significant difference in carriage of *Candida* isolated between betel quid chewers and non-chewers. In contrast, our study observed the association of being betel quid chewers and high levels of *Candida*. This is probably due to the fact that our study separated the level of *Candida* carriage into the low and the high groups as opposed to use only presence or absence of *Candida* as in other studies. If only one level of *Candid*a was determined, the association between *Candida* carriage and being current betel quid chewers might be altered. Since both betel quid chewing habit and the presence of *Candida* in the OPMDs could have a synergistic effect in promoting malignant transformation of OPMDs, the participants with betel quid chewing habit and high *Candida* levels should be robustly monitored to prevent malignant transformation.

In the present study, it was found that the use of dentures also increased risk of having high levels of *Candida* by 1.96 times. Prolonged denture wearing, poor denture hygiene, and mucosal trauma are important local factors that contribute to increased levels of Candida as a breach in the oral epithelium creates a portal of entry of *Candida* [26]. Similar to our study, it was also found that in Japan the use of dentures was significantly associated with the colonization of non-albicans (OR = 4.02; 95%CI 1.05–15.4).

Regarding hyposalivation, patients with hyposalivation were at 2 times more likely to have high levels of *Candida* as compared to those with normal salivation. More than half of the participants had decreased saliva production without any feelings of mouth dryness. It is worth pointing out that the prevalences of xerostomia (43.5%) and hyposalivation (64.1%) observed in our study were relatively high. This might be due to the fact that the majority of participants in the present study were elderly people and they were more likely to have dry mouth. Similar to a study by Torres et al. [16], a significant correlation between salivary flow rates and *Candida* colony counts in the saliva of patients with xerostomia was reported. Saliva is enriched with antimicrobial proteins that aids in elimination of *Candida* from the oral cavity. Therefore, quantitative and qualitative reductions of saliva are common factors implicate for the high counts of *Candida.*

One more risk factor that associated with the presence of high *Candida* levels was being in the CSOC group. The oral lesions presented in the CSOC group were pseudomembranous and erythematous candidiasis, angular cheilitis, medial rhomboid glossitis and *Candida* leukoplakia. Some of the participants presented with atrophic glossitis with discomfort and changes of taste perception which indicated oral candidiasis. The mean score from the dip-slide test in the CSOC group was highest (2.39 ± 1.44) which supported a diagnosis of oral candidiasis (Table 2). The privilege of having oral medicine specialists and oral surgeons in this screening was that the lesions clinically diagnosed with candidiasis can be treated promptly and this could help improvement of the overall health of the elderly participants enrolled in this study.

In addition, participants in the CSOC group had lowest salivary flow rate which might render a high level of oral *Candida* (Table 1). Additionally, the participants in this cohort were elderly people living in the northeastern area and a lot of them had thalassemia, a predominant type of anemia often found in the people living in the northeastern area of Thailand [27]. It is obvious that thalassemia can have a significant influence on the immune system and can render increase *Candida* overgrowth in the oral cavity. Prompt treatment was given in this group of participants including antifungal therapy, increase hydration, stimulation of saliva production and close follow up of the participants after antifungal treatment. Regular check up every 3–6 months was also encouraged in this group of participants so that the oral candidiasis could be monitored.

We further investigated whether any of the questions in the xerostomia questionnaire would suggest that patients might have high levels of *Candida* in the oral cavity. Significantly high number of participants who answered yes in the question “Do you have problems in tasting food?” had high levels of *Candida.* The answer could be due to the fact that the participants’ mouth was dry or high levels of *Candida* could contribute to the changes in oral taste perception. Further study may be needed to evaluate the changes of perception in participants with high levels of *Candida.*

The main strength of this study is its relatively large sample size. The recruited participants were those attended one of the largest OPMDs and OC program in Thailand by now. In addition, as a result of having a large study population, a variety of oral lesions including OPMDs, non-OPMDs as well as OC, were investigated in our study. Although the participants had been screened by dental hygienists and general dentists for OPMDs and OC at the sub-district hospital, a high number of participants in the NOM group (n = 132) was found. This might be due to the fact that at the time when dental hygienists and general dentists screened for oral lesions, there might be some oral lesions that were caused by chronic irritation. The dental hygienists and general dentists were trained to eliminate any irritations in the oral cavity that can cause these lesions prior to the referral to district hospitals. Therefore, at the time when participants attended the district hospitals, the lesions resolved and the oral cavity appeared normal. In addition, there might be some normal variations in the oral cavity that dental hygienists and general dentists noted as suspicious oral lesions. Therefore, the dental hygienists and general dentists referred those participants with normal variation to assure that these lesions were not harmful.

We also acknowledged the limitation in this study. Although it was expected that the presence of high *Candida* levels would be associated with the occurrence of OPMDs and OC we did not find such association in this study. The majority of the participants in this study were elderly people, therefore, travelling back to the district hospital for the follow up was a major limitation in this study. In addition, the study was conducted during the COVID-19 pandemic period and this rendered more difficulty in the follow up in participants that had OPMDs/OC. However, since Thailand had an effective VHV system, these VHVs might be able to facilitate the follow up of most of the participants at their homes.

## Conclusion

This study indicated that 31.3% of participants with high levels of *Candida* were in the OPMDs/OC group. Factors associated with high levels of *Candida* were ages above 60 years old, female gender, being present betel quid chewer, use of denture, hyposalivation and being in the CSOC group. Comprehensive and multidisciplinary management should be given especially for individuals in the OPMDs/OC and the CSOC groups which would lead to a significant improvement of the overall good health.

## Data Availability

The datasets used and/or analyzed during the current study are not publicly available due the confidentiality of the participants but are available from the corresponding author on reasonable request.

## List of abbreviations

NOM: Normal oral mucosa
OPMDs: Oral potentially malignant disorders
OC: Oral cancer
CSOC: Clinically suspected oral candidiasis
HIV: Human immunodeficiency virus
VHVs: Village health volunteers
GLOBOCAN: Global Cancer Observatory
IARC: The International Agency for Research on Cancer
WHO: World Health Organization
ml: Milliliter
CFU: Colony forming unit
SD: Standard deviation
OR: Odds ratio
CI: Confidence interval
